# Fragilitas Ossium

**Published:** 1861-11

**Authors:** Orrin Smith

**Affiliations:** Chicago, Ill.


ARTICLE II.

### FRAGILITAS OSSIUM.

By ORRIN SMITH, A. M., M. D., Chicago.

A Paper, read at the October Meeting of the Chicago Medical Society, Oct. 18th, 1861.

June 16, 1860.—I was called to see Mrs. G. W. Field, living on North Wells street, aged 40, who had been suffering some years from scirrhus of both mammæ and the glands in the right axilla ; the physical signs and symptoms also denoted scirrhus deposit in both lungs, and in the mesenteric glands. The respiration was hurried, to some extent, the respiratory murmur considerably obscured over most of both lungs, with dullness on percussion, and a small secretion of frothy mucus, which did not at any time become abundant. She had a very constant irritative cough, not relieved by expectorants, and only moderately so by anodynes and stimulants, of which she made but a very moderate use. The stomach, for some months after my first visit, digested light food, in moderate quantities, without a great amount of suffering;—still nutrition was never well performed after I saw her, and she continued to lose flesh and strength gradually, the blood-vessels carrying only a moderate amount of blood at any time subsequently. Her pulse was small, irritable and quick; tongue slightly covered with white coat. She had considerable tenderness over the region of the mesentery, and most of the time, an uneasy, painful sensation. As emaciation progressed, enlarged mesenteric glands could be felt through the thin integuments of the abdomen. There was soreness and swelling of the joints of both hips, and some in the knees, accompanied with much pain, and all the usual phenomena of sub-acute rheumatism, which soon yielded to appropriate treatment, although she was never, after I first saw her, entirely free from pain and tenderness along the sciatic nerve. The spinal cord and the muscles along the spine, also suffered, at intervals, from this rheumatic condition, during the remainder of her life—so much so, that from pain, lameness, attenuation and consequent weakness, she was unable to walk without the assistance of friends, or supporting herself with her hands. The bowels were easily kept soluble by properly regulated diet; indeed, were more inclined to be too loose than otherwise, until the two last months of her life,—as in all cases where diseased mesenteric glands are interposed between the functions of digestion and nutrition. The emaciation became so extreme, that not only the fatty matters were re-absorbed, but the other animal tissues were called upon to that degree, that she distinctly felt and appreciated the want of the animal tissues in the bones, and frequently cautioned her assistants to move her carefully, or they would break her bones,—she, evidently, feeling a want of tenacity in these structures.

Two days before her death, while in a paroxysm of coughing,—a paroxysm not characterized by great force,—while sitting, well supported and bolstered in her chair, she broke the right femur, with a transverse fracture at about the upper third, by the contraction of the muscles, aided, perhaps, by an uneven pressure of the pillow upon which she sat, although she was not conscious of any such inequality. After the fracture, she remained sitting in her chair until I arrived to dress her limb,—a period of about an hour—when I found the broken ends of the bone in contact, and elevated to an angle of about forty-five degrees, making a great deformity. The fracture gave but little or no shock to the system, and was not very tender or painful, and of course not followed by any amount of re-action. It was dressed in the usual way, with the many-tailed bandage and splints. The muscles of the thigh were so attenuated, that after the application of the bandage, the limb more resembled in size, a small arm than a thigh. The limb was well secured by splints, so that it could be moved without danger of misplacement, as her difficulty of breathing and necessities might require, and then rested on a double-inclined plane, and the friends instructed to remove and re-adjust the same as she might desire,—the object being the patient's comfort, no union of the fracture being expected. Her principal sufferings at this time, and for months previous, were from the want of nutrition, and its consequent thirst, languor, and exhaustion, which were very great.

Two days after the fracture of the thigh, while sitting bolstered up in her chair, with a lady friend supporting her forehead with one hand, and the other over the upper dorsal vertebræ, during a paroxysm of coughing, a distinct snap, or fracture of the bones of the neck was felt by the attendant, and heard by her, and also by all in the room. Her respiration was instantly suspended, and they laid her on the bed; her pulse continued to beat, they judged, for the period of ten minutes, but there was no respiration.

It may also be added, that when the thigh was broken, the fracture was distinctly heard by all in the room. And she immediately said before any examination was made, that her thigh was broken. The little force which it took to fracture her bones, can be inferred by the want of muscular tissue, as before indicated. I did not see the body after death, until it was placed in the coffin, when, on examination, I found more than ordinary motion about the region of the two upper vertebræ of the neck, and have no hesitancy in believing that she died from compression of the spinal cord, consequent on a fracture of these bones, occurring August 1, 1861, a period of a little more than a year after I first saw her.

I am also informed by her husband, who is a man of the strictest truth and integrity, that a sister of hers died in June previous, with scirrhus of about the same length of time; and that a day or two before her death, while lying in bed, she broke both femurs, simultaneously, near their union with the bones of the pelvis, during a fit of coughing. Her history, as I received it from the above authority, would be very nearly a transcript of this paper.

